# *In silico* analysis of SARS-CoV-2 genomes: Insights from SARS encoded non-coding RNAs

**DOI:** 10.3389/fcimb.2022.966870

**Published:** 2022-11-28

**Authors:** Neha Periwal, Urvashi Bhardwaj, Sankritya Sarma, Pooja Arora, Vikas Sood

**Affiliations:** ^1^ Department of Biochemistry, Jamia Hamdard, New Delhi, India; ^2^ Department of Zoology, Hansraj College, University of Delhi, Delhi, India

**Keywords:** coronavirus, SARS-CoV-2, miRNAs, non-coding RNAs, SARS

## Abstract

The recent pandemic caused by Severe Acute Respiratory Syndrome Coronavirus-2 has resulted in enormous deaths around the world. Clues from genomic sequences of parent and their mutants can be obtained to understand the evolving pathogenesis of this virus. Apart from the viral proteins, virus-encoded microRNAs (miRNAs) have been shown to play a vital role in regulating viral pathogenesis. Thus we sought to investigate the miRNAs encoded by SARS-CoV-2, its mutants, and the host. Here, we present the results obtained using a dual approach i.e (i) identifying host-encoded miRNAs that might regulate viral pathogenesis and (ii) identifying viral-encoded miRNAs that might regulate host cell signaling pathways and aid in viral pathogenesis. Analysis utilizing the first approach resulted in the identification of ten host-encoded miRNAs that could target the SARS, SARS-CoV-2, and its mutants. Interestingly our analysis revealed that there is a significantly higher number of host miRNAs that could target the SARS-CoV-2 genome as compared to the SARS reference genome. Results from the second approach resulted in the identification of a set of virus-encoded miRNAs which might regulate host signaling pathways. Our analysis further identified a similar “GA” rich motif in the SARS-CoV-2 and its mutant genomes that was shown to play a vital role in lung pathogenesis during severe SARS infections. In summary, we have identified human and virus-encoded miRNAs that might regulate the pathogenesis of SARS coronaviruses and describe similar non-coding RNA sequences in SARS-CoV-2 that were shown to regulate SARS-induced lung pathology in mice.

## Introduction

Coronaviruses are a group of enveloped viruses with positive-sense single-stranded RNA as the genetic material. These viruses cause mild to severe respiratory and intestinal infections in immune-compromised individuals. They were not considered to be pathogenic until the outbreak of Severe Acute Respiratory Syndrome (SARS) in China and the Middle East Respiratory Syndrome Coronavirus (MERS-CoV) in Middle East countries in 2002 and 2012 respectively when the viral infection led to significant deaths among the infected individual’s (Song et al., 2019). As on 24 September 2022, the SARS-CoV-2 pandemic has resulted in more than 611 million infections resulting in 6.5 million deaths globally (https://covid19.who.int/). Data from the ongoing pandemic suggest that mortality caused by this virus has already surpassed that of combined mortality caused by both SARS and MERS coronaviruses. Though SARS-CoV-2 has caused significant mortality and morbidity, its evolution trajectory points towards an increased transmissibility (Volz et al., 2021). SARS-CoV-2 has a slow evolutionary rate i.e. 1 or 2 nucleotide alterations/month/lineage in the viral genome which consists of around 30,000 base pairs (Boehm et al., 2021). Despite this slow mutation rate, SARS-CoV-2 has evolved into around sixteen lineages in a short time since the onset of the pandemic (Tegally et al., 2021). The underlying mutations in these lineages allow the virus to transmit efficiently thereby enhancing the viral fitness. The random mutations in the viral genomes have led to changes in the viral proteins resulting in improved functions (Korber et al., 2020). However, apart from modulating the functions of the viral proteins, the mutations in the viral genomes can lead to changes in the microRNA binding sites thereby resulting in the appearance of the escape mutants (Hosseini Rad and McLellan, 2020).

MicroRNAs (miRNAs) are non-coding RNA molecules that are ~21 nucleotides long and can regulate gene expression primarily *via* imperfect base pairing with the 3’ untranslated regions of the target RNA molecules (Duchaine and Fabian, 2019; Liu and Wang, 2019). Apart from regulating the biological pathways, miRNAs have been shown to modulate viral infections (Mishra et al., 2020). Following viral infections, innate immune responses are induced leading to the generation of antiviral responses. This arm of antiviral signaling constitutes several potential antiviral factors including antiviral miRNAs. These miRNA molecules can lead to the inhibition of viral replication. For instance, miR-196, miR-351, and miR-448 have been shown to inhibit the Hepatitis C Virus infection (Pedersen et al., 2007). The miR-155 has been shown to regulate type I interferon signaling thereby modulating the replication of several viruses including HIV-1 (Swaminathan et al., 2012), Epstein-Barr virus (Jiang et al., 2006), reticuloendotheliosis virus (Bolisetty et al., 2009), Dengue virus (Su et al., 2020), West-Nile virus (Natekar et al., 2019), Japanese encephalitis virus (Thounaojam et al., 2014; Pareek et al., 2014) and Herpes Simplex Virus 1 (Wang et al., 2019). Conversely, viruses have also been shown to encode small non-coding RNA molecules to facilitate viral replication. It has been shown that several miRNAs encoded by Kaposi’s Sarcoma-associated Herpesvirus help in maintaining the viral latency (Lei et al., 2012). Several RNA viruses including Influenza virus (Li et al., 2018), HIV-1 (Zhang et al., 2014; Li et al., 2016), Dengue virus (Hussain and Asgari, 2014), and Ebola virus (Prasad et al., 2020) are also shown to encode small non-coding RNAs which can modulate the viral infection. Additionally, a complex cross-talk among miRNA and host factors further contributes to the replication of the viruses in their host (Bruscella et al., 2017; Mishra et al., 2020).

The ongoing SARS-CoV-2 pandemic has driven several studies to identify miRNAs as alternative therapeutics against the virus (Panda et al., 2022). Studying miRNAs has become crucial for the development of possible diagnostics, prognostics and therapeutics that could help in treatment strategies in the COVID-19 (Fani et al., 2021; Ying et al., 2021; Ahmed et al., 2022). Several studies aiming to predict miRNAs regulating SARS-CoV-2 pathogenesis were published and various miRNAs are identified that might have an impact on the replication of SARS-CoV-2 (Chen and Zhong, 2020; Marchi et al., 2021; Kucher et al., 2022; Morales et al., 2022; Yang et al., 2022). Apart from directly targeting the viral genomes, the miRNAs can indirectly regulate viral replication *via* regulating host factors that are critical for viral replication. The expression of proteins implicated in the SARS-CoV-2 life cycle, including ACE2, TMPRSS2, Spike proteins, and Nsp12, can be inhibited by miRNAs (Yang et al., 2022; Paul et al., 2022). The type I Interferon pathway is inhibited by SARS-CoV-2 encoded miRNAs, which also regulate the allelic differential expression of vulnerable genes (Zhu et al., 2021). Additionally, it has been shown that miRNAs can regulate SARS-CoV-2 replication *via* modulating certain receptors including ACE2, AXL, ADAM17, HAT1, NRP1, VMP1, Cyclophilin A, and Cathepsin B (Zhang et al., 2021; Zeng et al., 2022).

Numerous host- and virus-specific indicators linked to SARS CoV-2 infection have recently emerged (Sevgin and Sevgin, 2021). Host miRNAs were found to target ACE2 and TMPRSS2 genes which regulate SARS-Cov-2 cellular entry (Kaur et al., 2021; Bellae Papannarao et al., 2022) and can also be modified to act in a pro-viral manner by blocking host immune systems (Sevgin and Sevgin, 2021). Recently several host-encoded miRNAs were found to regulate ORF1a/b which is necessary for viral replication and translation (Arghiani et al., 2021). A study on SARS coronavirus successfully characterized the role of viral encoded non-coding RNAs in the virus-induced lung pathology (Morales et al., 2017). Furthermore, phylogenetic and taxonomy studies have confirmed that SARS-CoV-2 forms a sister clade with SARS coronavirus suggesting that rich information on SARS coronavirus can be leveraged to gain insights into SARS-CoV-2 (The species Severe acute respiratory syndrome-related coronavirus, 2020). It was observed that inhibiting viral encoded non-coding RNAs led to a reduction in the lung pathology of virus-infected mice. Taking into consideration the significant similarity between SARS and SARS-CoV-2 and the role of non-coding RNAs in virus pathogenesis and cell signaling pathways, we aimed to study host and viruses encoded miRNAs and compare them among SARS, SARS-CoV-2, and the mutants to understand the pathogenesis of these viruses.

In this study, we utilized a suite of highly popular algorithms to identify host and virus-encoded miRNAs that might have the potential to regulate SARS-CoV-2 pathogenesis. Moreover, we compared our results along with data from other published studies to identify a common set of miRNA candidates. These high-confidence candidates can then be validated in the wet lab as well as provide us with the opportunity to design broadly acting drug molecules against SARS-CoV-2 and its mutants.

## Methods

### Identification of host miRNAs targeting several SARS-CoV-2 and SARS reference genomes

MicroRNAs are known to regulate diverse biological pathways including various steps in viral replication (Bernier and Sagan, 2018). Hence, we studied the role of host miRNAs in regulating viral replication by predicting the host miRNA binding sites among the genome of these viruses. In order to gain an in-depth understanding of the pathogenesis of SARS-CoV-2, we analyzed the reference genome of SARS-CoV-2 along with its variants namely Alpha (B1.1.7), Delta (B.1.617.2, AY.4.2), Beta (B.1.351), Omicron (B.1.1529, BA.1, BA.3, BA.1.1), Gamma lineage, Epsilon (B.1.27), Eta (B.1.525), Kappa (B.1.525), Lambda (C.37), Mu (B.1.627) and Theta (P.3) that were obtained from NCBI (Brister et al., 2015) and GISAID (Khare et al., 2021) (**Supplementary table 1**). The SARS reference genome was used for the comparison and the analysis was performed on both the forward and reverse sequences of viral genomes. For the prediction of host miRNAs that could bind to viral genomes, the miRDB algorithm was used (Chen and Wang, 2020). The analysis was performed in the custom mode where the user-defined sequences could be uploaded and processed. All the analyses were done on default settings and only those miRNAs were studied further that had a target score >= 80 so as to follow stringent selection criteria. Thus we obtained a list of miRNAs that could potentially target SARS-CoV-2 and its mutants. Recently, several studies were published to predict host-encoded miRNAs that might regulate SARS-CoV-2. Hence, we present the common miRNAs that are predicted by multiple studies involving diverse set of prediction algorithms.

### Identification of SARS-CoV-2 and SARS encoded miRNAs

Several viruses have been shown to regulate host signaling pathways by encoding miRNAs in their genomes. However, there have been debates as to whether RNA viruses could encode miRNAs or not. Recent studies have successfully identified several miRNAs that are encoded by various RNA viruses including HIV-1 (Bennasser et al., 2004), West Nile Virus (Hussain et al., 2012), Dengue (Hussain and Asgari, 2014), Influenza (Li et al., 2018) and Ebola virus (Prasad et al., 2020). It was also observed that genomic sequences of these viruses could fold into stem-loop structures which gave rise to miRNAs (Klaver and Berkhout, 1994). Hence, in order to gain insights into various miRNAs possibly encoded by genomes of SARS coronavirus, SARS-CoV-2, and its variants, a set of computational tools was used to predict miRNAs encoded by these viruses. Since hairpin loops have been shown to produce miRNAs in RNA viruses, we used the miRNAFold algorithm to predict hairpin loops in viral genomes (Tempel and Tahi, 2012). As all hairpin loops do not result in functional miRNAs, hence output file from the miRNAFold algorithm was further processed using miRBoost algorithm to identify whether the predicted hairpin structures could form microRNAs or not (Tran et al., 2015). Once the viral encoded miRNAs were identified, the results were then processed using miRBase to find any similarities among the virus-encoded miRNAs and human miRNAs (Kozomara et al., 2019).

### Comparison of SARS encoded non-coding RNA sequences with SARS-CoV-2 genomes

Recently, deep sequencing based approaches identified SARS encoded non-coding RNAs which played a critical role in virus-induced lung pathology (Morales et al., 2017). In order to gain an understanding of the genomic regions responsible for the generation of non-coding RNAs, we aligned SARS-CoV-2 sequences along with the mutants SARS reference sequence using the Clustal omega (Sievers et al., 2011). Aligned SARS genomic sequences that were shown to encode non-coding RNAs were then further studied to identify the percentage homology with that of SARS-CoV-2 and its mutants.

## Results

### Identification of host miRNAs targeting several SARS, SARS-CoV-2 and its variants

**Figure 1:**
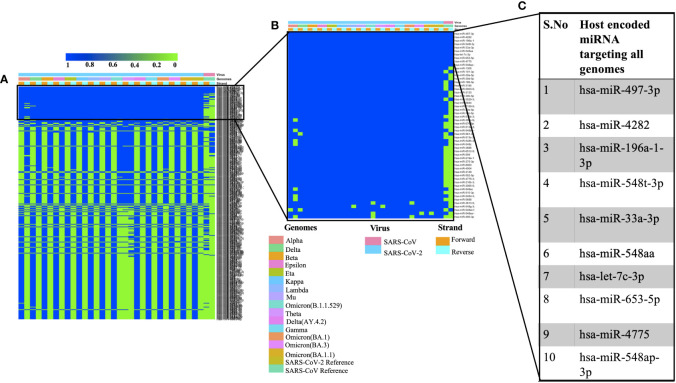
Shows the host-encoded miRNAs that could target SARS, SARS-CoV-2, and its variants. **(A)** Common miRNAs that could target 45% of genomes from the study. **(B)** Host miRNAs that could target 80% of the genomes from the study and **(C)** Top 10 miRNAs targeting all the forward and reverse genomes used in this study.

We used miRDB algorithm to gain insights into host encoded miRNAs (*Homo sapiens*) that could target viral genomes. The analysis was performed on both the forward and reverse genomic sequences of all the genomes that were being studied. Our analysis identified several host miRNAs that could target each of the forward and reverse genomic sequences of SARS, SARS-CoV-2 and its variants (**Supplementary Table 2**). **Figure 1A** illustrates the common host miRNAs (n=354) that were predicted in 45% of the genomes used in this study. Similar analysis of common host miRNAs targeting 80% of the genomes identified 54 miRNAs (**Figure 1B**). A comparison of all the forward and reverse sequences of SARS, SARS-CoV-2 and its variants revealed 10 host miRNAs that could target all the genomes being studied and hence could be potentially used as broad-acting antivirals (**Figure 1C**). Out of these 10 miRNAs, three of them including hsa-miR-4282, hsa-miR-33a-3p and hsa-miR-4775 were found in several other studies. The hsa-miR-4282 has been shown to target ATF2 gene (Vadivalagan et al., 2022) that arrests the viral infection, hsa-miR-33a-3p targets SMAD4 which is involved in the Wnt signalling pathway (Yousefi et al., 2020), similarly hsa-miR-4775 targets SMAD7 and it plays role in TGF-beta signaling pathway (Yousefi et al., 2020). The hsa-miR-497-3p, inhibits vascular endothelial growth factor A, suppresses angiogenesis, and is found to be involved in the body’s defense mechanisms (Van Laar et al., 2018). Another miRNA, hsa-miR-196a-1-3p was predicted to be able to bind to SARS-CoV, MERS-CoV, and SARS-CoV-2 genomes (Kim et al., 2020). The hsa-miR-548t-3p was found to be down-regulated in the ischemic stroke (Li et al., 2022) whereas hsa-miR-33a-3p was shown to have ~90% sequence identity with VARV and VACV viral genomes. It was observed that hsa-miR-33a-3p targets A46L and A41L genes respectively thereby modulating the host defense mechanisms (Hasan et al., 2016). Recently, other groups have also utilized a diverse set of algorithms to identify the miRNAs that could target SARS-CoV-2 genomes (Mosharaf et al., 2022; Samy et al., 2022; Shirvani et al., 2022; Zeng et al., 2022). In order to further advance our understanding of host miRNAs targeting SARS-CoV-2 genomes, we compared our data with other studies. We observed several host-encoded miRNAs including hsa-miR-3529-3p, has-miR-516b-5p, and hsa-miR-155-5p that were common in many studies pointing toward the potential miRNAs that might be the strong candidates for further wet-lab validation and further understanding how these miRNAs are contributing towards the pathogenesis of SARS-CoV-2.

### Identification of miRNAs encoded by SARS, SARS-CoV-2 and its variants

**Figure 2:**
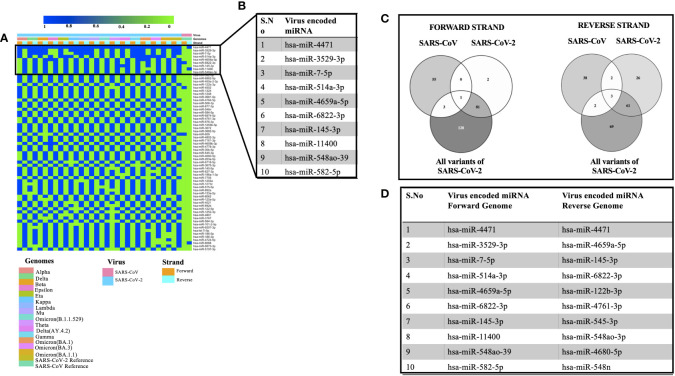
Showing the viral encoded miRNAs that had similarity with that of miRNAs encoded by the *H sapiens.* **(A)** Heatmap showing miRNAs encoded by 45% of the genomes under study. **(B)** Top 10 miRNAs that could target various genomes used in the study. **(C)** Common miRNAs among the forward and the reverse genomic sequences of SARS, SARS-CoV-2 and its variants. **(D)** Top 10 miRNAs targeting forward and the reverse genomes used in the study.

Viral encoded miRNAs have been shown to regulate cellular signaling pathways. RNA viruses are known to produce miRNAs that are critical for their replication cycle (Li and Zou, 2019). In order to identify miRNAs encoded by SARS, SARS-CoV-2, and its mutants, we utilized a suite of algorithms that could identify stem-loop structures in the viral genomes. This was followed by the prediction of miRNAs among these stem-loop structures and finally comparing the predicted miRNAs with that of human-encoded miRNAs. As mentioned earlier, the miRNAFold algorithm was used to predict the stem loop structures in the viral genomes. The algorithm predicted various hairpin loops in genomes of SARS, SARS-CoV-2, and its variants. We used both the forward and the reverse strand of viral RNA to predict the stem-loop structures. Since all the predicted stem-loop structures do not form miRNAs, we used the miRBoost algorithm to identify potential stem-loop structures that could form miRNAs. The results thus obtained were then processed with the miRBase algorithm to investigate any similarity among virus and host-encoded miRNAs. We used this step with the rationale that any similarity among viral and host-encoded miRNAs will lead to the modulation of similar signaling pathways. The miRbase algorithm identified several viral encoded miRNAs that had similarities with human encoded miRNAs (**Supplementary Table 3**). **Figure 2A** represents the miRNAs that were predicted in 45% of the genomes used in this study. **Figure 2B** presents the top 10 miRNAs that can target various coronavirus genomes used in this study. Comparative analysis of miRNAs predicted from all the genomes used in this study revealed that hsa-miR-4471 was present in 33 out of 34 genomes. This miRNA was predicted to be encoded by SARS-CoV-2 and all its mutants including the forward and the reverse genomic sequences. It was further observed that hsa-miR-4471 was encoded by only the reverse sequence of SARS coronavirus but not by the forward sequence.

A literature survey for the top ten miRNAs targeting SARS, SARS-CoV-2, and its mutants revealed that some of the miRNAs have been shown to play a key role in coronavirus pathogenesis. For instance, hsa-miR-3529-5p could modulate SARS-CoV-2 by targeting NF-kb pathway whereas hsa-miR-7-5p was shown to modulate cellular apoptosis *via* mTOR pathway (Abedi et al., 2021), hsa-miR-145-3p could regulate SARS-CoV-2 *via* interfering with SMAD3 levels and can also target 5’ end of the Ebola virus genome (Golkar et al., 2016) and hsa-582-5p could target ACE-2 which is an important receptor of SARS-CoV-2 (Kim et al., 2020). Comparative analysis of miRNAs targeting all the forward and reverse genomic sequences led us to the identification of hsa-miR-4502 as a miRNA common in all the forward genomic sequences of SARS, SARS-CoV-2, and its mutants. Since these miRNAs are encoded by viral genomes, the prediction of a single miRNA in all the forward genomes of SARS-SARS-CoV-2 and its mutants points toward the importance of the putative miRNA. Our results are in agreement with the previous study that identified similarities among SARS-CoV-2 encoded pre-miRNAs and human encoded hsa-miR-4502 (Aydemir et al., 2021). Analysis of all the reverse genomic sequences of SARS, SARS-CoV-2 and its mutants led us to the identification of three miRNAs (hsa-miR-8088, hsa-miR-11400, hsa-miR-4471) were found common in all reverse sequences (**Figure 2C**). The presence of these miRNAs in the reverse sequences of all the viral genomes under study points toward their importance in the viral life cycle.

Then we compared the miRNAs targeting forward and reverse genomes of all the SARS-CoV-2 variants used in the study (**Figure 2D**). It was observed that some of the predicted miRNAs could target cellular host factors thereby facilitating the replication of SARS-CoV-2. Some of the miRNAs targeting forward genomes of SARS-CoV-2 variants included hsa-miR-3672, hsa-miR-7161-5p, hsa-miR-30b-5p and hsa-miR-3682-5p were have been shown to regulate host factors including ICOSLG, G3BP1/P2, CREB1 respectively (Vastrad et al., 2020; Almutairy et al., 2021; Vadivalagan et al., 2022). Analysis of reverse sequences of SARS-CoV-2 mutants revealed that has-miR-548 could regulate G3BP1/P2 thereby contributing to viral replication (Almutairy et al., 2021).

### Comparison of SARS encoded non-coding RNA sequences with SARS-CoV-2 and its mutants

**Figure 3:**
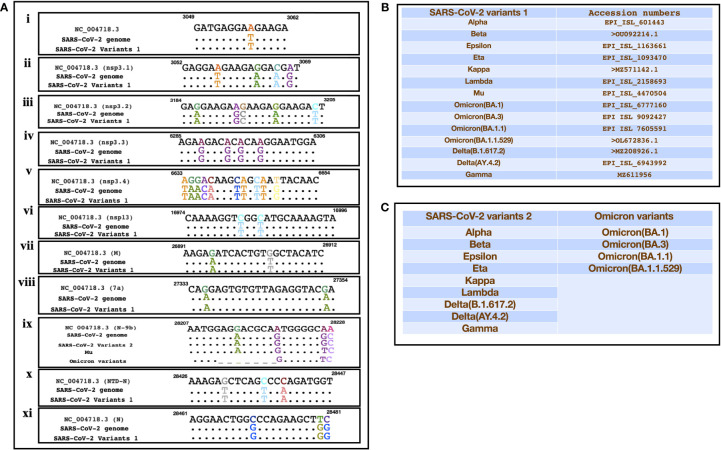
Showing the alignment of genomic regions of SARS-CoV-2 and its mutants with those regions of SARS that were shown to produce non-coding RNAs. **(A)** (i-xi)) represents the genomic regions where SARS encoded ncRNAs were identified (Morales et al., 2017). **(B, C)** Represent the sequences of SARS-CoV-2 variants that were used for the comparison.

A recent study had identified SARS encoded non-coding RNAs that contributed towards the pathology of the virus (Morales et al., 2017). In an attempt to understand the pathogenesis of SARS-CoV-2 and its variants, we compared its genomic sequence with that of SARS to estimate the sequence similarity among both the viruses in genomic regions that resulted in non-coding RNAs. We observed a very similar “GA” rich region SARS-CoV-2 including its variants that was reported to be enriched in several SARS encoded non-coding RNAs. This region was found to be highly conserved in all the variants except the omicron variant (BA.1, BA.3, BA.1.1, B.1.1.529) which had a deletion in N-9b domain from position 28038-28047 [**Figure 3A**(ix)]. Furthermore, comparative analysis for other non-coding RNAs revealed that SARS-CoV-2 also had similar stretches albeit with some nucleotide changes (**Figure 3A**). **Figures 3B, C** represents the SARS-CoV-2 variants that were used for the comparison. Interestingly genomic regions of SARS-CoV-2 had more than 85% similarity with SARS encoded scRNA-N whose inhibition led to a reduction in SARS-induced lung pathology. Apart from scRNA-N, SARS-CoV-2 showed a high degree of similarity (>80-90%) with several SARS encoded non-coding RNAs from regions Nsp3.3, Nsp13, Membrane protein, 7a, N-9b, N-TDN, and Nucleocapsid protein. This high level of similarity among SARS coronavirus encoded non-coding RNAs with that of SARS-CoV-2 genomes suggest that the virus might also encode similar non-coding RNAs that have been shown to contribute towards lung pathology.

## Discussion

The recent pandemic of COVID-19 has caused significant mortality around the world. Since its origin in Wuhan, China SARS-CoV-2 has been able to spread throughout the world in a very short duration. The efforts of the scientific community to design effective drugs against the virus are hindered by the emergence of SARS-CoV-2 mutants. The virus has evolved into more than sixteen lineages in a very short span of time. Therefore, understanding the evolution of the virus is of utmost importance. Scientists are racing against the time to find an effective cure for the virus. Apart from regulating vital cellular processes (Andergassen and Rinn, 2022), non-coding RNA has been shown to regulate viral pathogenesis (Liu et al., 2019) and they act as a negative regulator and inhibit antiviral responses (Kesheh et al., 2022). It has also been stipulated that the SARS-CoV-2 genome has sequence similarity with the host non-coding RNAs thereby resulting in the possible epigenetic crosstalk among the virus and its host (Talotta et al., 2022). lncRNAs that were found to be functionally linked and implicated in the innate immune response are upregulated as a result of SARS-CoV-2 infection (Enguita et al., 2022). They were found to alter the host’s immune response (Paul et al., 2022). Therefore, the present study was designed to understand the dynamics of non-coding RNA among SARS, SARS-CoV-2, and its mutants. We used a suite of highly popular algorithms to identify host and viral encoded miRNAs that might affect viral replication in host cells. Owing to the significant similarity of SARS-CoV-2 with that of SARS, we sought to leverage the knowledge generated on SARS genomic sequences and apply it to SARS-CoV-2 and its mutant sequences to identify possible overlaps among both the viruses as well as understand ncRNA dynamics as the virus evolves. We compared several SARS-CoV-2 viral genomic sequences and their mutants with that of the SARS reference sequence in order to understand how they might be targeted by host-encoded miRNAs. Interestingly our data suggested that SARS-CoV-2 genomic sequences were targeted by significantly more numbers of host-encoded miRNAs as compared to the SARS reference genome. This might provide a possible explanation as to why the virus causes mild disease in healthy individuals. Moreover, our comparative analysis of our candidates with that of other published candidates led to the identification of ten host-encoded miRNAs that were identified in many studies. Further wet lab validations are required for the proper validation of these potential candidates. These viral encoded miRNAs have been shown to arrest the transition of phases in cell cycle by preventing the replication of specific genes thereby contributing towards the viral replication. For instance, Let-7c-3p which is one of the top ten miRNAs from our study has been shown to be associated with SARS-CoV-2 infection and pathogenesis (Jafarinejad-Farsangi et al., 2020).

Viruses are known to encode miRNAs to dampen the host responses. We utilized a suite of algorithms to identify possible stem-loop structures in viral genomes which could have the potential to be converted into viral encoded miRNAs. Our tools successfully identified several virus-encoded miRNAs that had significant similarities with host miRNAs. Viral encoded miRNAs having very similar sequences to host-encoded miRNAs might be an excellent strategy to regulate cell signaling pathways. Though we identified several miRNAs encoded by SARS and SARS-CoV-2, despite the high degree of genomic similarity among SARS and SARS-CoV-2, we could not identify common miRNAs among both of them.

Recently, SARS-encoded non-coding RNAs were identified in the lungs of infected mice. These non-coding RNAs were distributed along the entire viral genome and antagomir-based inhibition of these viral encoded RNAs resulted in reduced lung pathology. This data indicate an important role of these viral encoded RNAs during viral pathogenesis. Therefore, we aligned all the SARS-CoV-2 and mutant genomic sequences with the SARS reference genome to investigate whether non-coding RNA sequences among both the viruses are conserved or not. We observed that there was a very similar “GA” rich region in all SARS-CoV-2 genomes including the mutants, that was identified as a part of SARS encoded non-coding RNAs. We also found a very high similarity among SARS encoded non-coding RNAs and all SARS-CoV-2 and mutant genomic sequences suggesting a possible role of these non-coding RNAs in SARS-CoV-2 pathogenesis.

To conclude, we have identified potential host encoded miRNAs that could target genomic sequences of SARS, SARS-CoV-2 and its mutants. We have also identified virus-encoded non-coding RNAs that might modulate the pathogenesis of SARS-CoV-2 thereby identifying new molecular targets against the virus.

## Data availability statement

The original contributions presented in the study are included in the article/**Supplementary Material**. Further inquiries can be directed to the corresponding author.

## Author contributions

VS and PA conceived the idea, designed the experiments, supervised the study wrote and finalized the manuscript. NP, UB, and SS performed the analysis. All authors contributed to the article and approved the submitted version.

## Funding

NP is supported by UGC fellowship. VS is supported by UGC under FRP programme. The study was funded by startup grant to VS by UGS and DST PURSE grant.

## Acknowledgments

We are thankful to all those scientists who contributed to SARS-CoV-2 genomic sequences.

## Conflict of interest

The authors declare that the research was conducted in the absence of any commercial or financial relationships that could be construed as a potential conflict of interest.

## Publisher’s note

All claims expressed in this article are solely those of the authors and do not necessarily represent those of their affiliated organizations, or those of the publisher, the editors and the reviewers. Any product that may be evaluated in this article, or claim that may be made by its manufacturer, is not guaranteed or endorsed by the publisher.
