# Supplementary material for: In silico analysis of SARS-CoV-2 genomes: Insights from SARS encoded non-coding RNAs
Source: Front Cell Infect Microbiol. 2022 Nov 28;12:966870. doi: 10.3389/fcimb.2022.966870 (PMC9742375; doi:10.3389/fcimb.2022.966870)
Supplement: Supplementary file 1 [file Table_1.docx]

**Supplementary Table 1:** Accession numbers of all genomes of SARS-CoV-2, SARS, and 15 variants of SARS-CoV-2.

| Genomes | Accession number |
| --- | --- |
| SARS-CoV-2 Genome | NC_045512.2 |
| SARS Genome | NC_004718.3 |
| SARS-CoV-2 Variants | OL672836.1 |
|  | MZ208926.1 |
|  | OU092214.1 |
|  | MZ368550.1 |
|  | MZ571142.1 |
|  | EPI_ISD_6943992 |
|  | EPI_ISL_1093470 |
|  | EPI_ISD_1172417 |
|  | EPI_ISD_7605591 |
|  | EPI_ISD_4470504 |
|  | EPI_ISD_1163661 |
|  | EPI_ISD_601443 |
|  | EPI_ISD_9092427 |
|  | EPI_ISD_6777160 |
|  | EPI_ISD_2158693 |

**Supplementary Table 2: Host encoded miRNAs present in genomes with their number of occurrences in each genome.**

| Host encoded miRNAs present in genomes | Number |
| --- | --- |
| hsa-miR-497-3p | 34 |
| hsa-miR-4282 | 34 |
| hsa-miR-196a-1-3p | 34 |
| hsa-miR-548t-3p | 34 |
| hsa-miR-33a-3p | 34 |
| hsa-miR-548aa | 34 |
| hsa-let-7c-3p | 34 |
| hsa-miR-653-5p | 34 |
| hsa-miR-4775 | 34 |
| hsa-miR-548ap-3p | 34 |
| hsa-miR-1305 | 33 |
| hsa-miR-101-3p | 33 |
| hsa-miR-30a-5p | 33 |
| hsa-miR-30d-5p | 33 |
| hsa-miR-186-5p | 33 |
| hsa-miR-3185 | 33 |
| hsa-miR-5003-5p | 33 |
| hsa-miR-3133 | 33 |
| hsa-miR-30b-5p | 33 |
| hsa-miR-3529-3p | 33 |
| hsa-miR-6844 | 33 |
| hsa-miR-7159-5p | 33 |
| hsa-miR-30e-5p | 33 |
| hsa-miR-30c-5p | 33 |
| hsa-miR-513b-3p | 32 |
| hsa-miR-302c-5p | 32 |
| hsa-miR-676-5p | 32 |
| hsa-miR-513a-3p | 32 |
| hsa-miR-548bb-3p | 32 |
| hsa-miR-561-3p | 32 |
| hsa-miR-513c-3p | 32 |
| hsa-miR-548h-3p | 32 |
| hsa-miR-548z | 32 |
| hsa-miR-3686 | 32 |
| hsa-miR-6512-5p | 32 |
| hsa-miR-559 | 32 |
| hsa-miR-219a-1-3p | 32 |
| hsa-miR-375-3p | 32 |
| hsa-miR-8063 | 32 |
| hsa-miR-4504 | 32 |
| hsa-miR-3149 | 32 |
| hsa-miR-552-5p | 32 |
| hsa-miR-4778-3p | 32 |
| hsa-miR-216b-3p | 32 |
| hsa-miR-3065-5p | 32 |
| hsa-miR-548ac | 32 |
| hsa-miR-510-3p | 32 |
| hsa-miR-548d-3p | 32 |
| hsa-miR-5688 | 31 |
| hsa-miR-3613-5p | 31 |
| hsa-miR-548g-3p | 31 |
| hsa-miR-548at-5p | 31 |
| hsa-miR-548as-3p | 30 |
| hsa-miR-495-3p | 29 |
| hsa-miR-421 | 24 |
| hsa-miR-6776-3p | 22 |
| hsa-miR-8068 | 21 |
| hsa-miR-4659a-3p | 20 |
| hsa-miR-4659b-3p | 20 |
| hsa-miR-122b-5p | 20 |
| hsa-miR-3606-3p | 19 |
| hsa-miR-599 | 19 |
| hsa-miR-144-3p | 19 |
| hsa-miR-5010-3p | 19 |
| hsa-miR-2053 | 19 |
| hsa-miR-382-5p | 19 |
| hsa-miR-592 | 19 |
| hsa-miR-1283 | 19 |
| hsa-miR-302b-5p | 19 |
| hsa-miR-302d-5p | 19 |
| hsa-miR-4801 | 18 |
| hsa-miR-8084 | 18 |
| hsa-miR-6507-5p | 18 |
| hsa-miR-4273 | 18 |
| hsa-miR-3168 | 18 |
| hsa-miR-7156-5p | 18 |
| hsa-miR-140-3p | 18 |
| hsa-miR-203a-3p | 18 |
| hsa-miR-429 | 18 |
| hsa-miR-452-5p | 18 |
| hsa-miR-5583-5p | 18 |
| hsa-miR-549a-3p | 18 |
| hsa-miR-892c-3p | 18 |
| hsa-miR-6809-3p | 18 |
| hsa-miR-200b-3p | 18 |
| hsa-miR-570-3p | 18 |
| hsa-miR-606 | 18 |
| hsa-miR-591 | 18 |
| hsa-miR-1208 | 18 |
| hsa-miR-5584-3p | 18 |
| hsa-miR-2054 | 18 |
| hsa-miR-548ad-3p | 18 |
| hsa-miR-4676-3p | 18 |
| hsa-miR-200c-3p | 18 |
| hsa-miR-10397-5p | 18 |
| hsa-miR-3129-3p | 18 |
| hsa-miR-5687 | 18 |
| hsa-miR-548ay-3p | 18 |
| hsa-miR-548at-3p | 18 |
| hsa-miR-433-5p | 18 |
| hsa-miR-888-5p | 18 |
| hsa-miR-3201 | 17 |
| hsa-miR-6505-3p | 17 |
| hsa-miR-934 | 17 |
| hsa-miR-3609 | 17 |
| hsa-miR-3611 | 17 |
| hsa-miR-12117 | 17 |
| hsa-miR-514a-3p | 17 |
| hsa-miR-3662 | 17 |
| hsa-miR-148b-5p | 17 |
| hsa-miR-1290 | 17 |
| hsa-miR-1537-3p | 17 |
| hsa-miR-4275 | 17 |
| hsa-miR-589-3p | 17 |
| hsa-miR-8056 | 17 |
| hsa-miR-4713-5p | 17 |
| hsa-miR-1260b | 17 |
| hsa-miR-508-3p | 17 |
| hsa-miR-2113 | 17 |
| hsa-miR-1252-3p | 17 |
| hsa-miR-571 | 17 |
| hsa-miR-4719 | 17 |
| hsa-miR-4999-3p | 17 |
| hsa-miR-6833-5p | 17 |
| hsa-miR-424-3p | 17 |
| hsa-miR-196b-5p | 17 |
| hsa-miR-4731-3p | 17 |
| hsa-miR-412-5p | 17 |
| hsa-miR-5187-3p | 17 |
| hsa-miR-21-3p | 17 |
| hsa-miR-3674 | 17 |
| hsa-miR-10523-5p | 17 |
| hsa-miR-3913-3p | 17 |
| hsa-miR-580-5p | 17 |
| hsa-miR-5680 | 17 |
| hsa-miR-3618 | 17 |
| hsa-miR-3167 | 17 |
| hsa-miR-466 | 17 |
| hsa-miR-196a-5p | 17 |
| hsa-miR-758-3p | 17 |
| hsa-miR-6755-3p | 17 |
| hsa-miR-3942-5p | 17 |
| hsa-miR-3646 | 17 |
| hsa-miR-4307 | 17 |
| hsa-miR-1245b-3p | 17 |
| hsa-miR-372-5p | 17 |
| hsa-miR-342-3p | 17 |
| hsa-miR-514b-3p | 17 |
| hsa-miR-188-3p | 17 |
| hsa-miR-4703-5p | 17 |
| hsa-miR-1911-5p | 17 |
| hsa-miR-4445-5p | 17 |
| hsa-miR-3682-5p | 17 |
| hsa-miR-1255b-2-3p | 17 |
| hsa-miR-4427 | 17 |
| hsa-miR-374c-5p | 17 |
| hsa-miR-876-5p | 17 |
| hsa-miR-655-3p | 17 |
| hsa-miR-142-3p | 17 |
| hsa-miR-6874-3p | 17 |
| hsa-miR-2681-5p | 17 |
| hsa-miR-4796-3p | 17 |
| hsa-miR-3692-3p | 17 |
| hsa-miR-4699-5p | 17 |
| hsa-miR-4666a-5p | 17 |
| hsa-miR-1238-3p | 17 |
| hsa-miR-548ah-5p | 17 |
| hsa-miR-4637 | 17 |
| hsa-miR-451a | 17 |
| hsa-miR-590-3p | 17 |
| hsa-miR-489-3p | 17 |
| hsa-miR-7849-3p | 17 |
| hsa-miR-377-3p | 17 |
| hsa-miR-511-5p | 17 |
| hsa-miR-12132 | 17 |
| hsa-miR-12120 | 17 |
| hsa-miR-3682-3p | 17 |
| hsa-miR-1260a | 17 |
| hsa-miR-4452 | 16 |
| hsa-miR-4698 | 16 |
| hsa-miR-4699-3p | 16 |
| hsa-miR-1226-3p | 16 |
| hsa-miR-505-3p | 16 |
| hsa-miR-3148 | 16 |
| hsa-miR-548w | 16 |
| hsa-miR-28-3p | 16 |
| hsa-miR-15b-5p | 16 |
| hsa-miR-4672 | 16 |
| hsa-miR-122b-3p | 16 |
| hsa-miR-503-5p | 16 |
| hsa-miR-410-5p | 16 |
| hsa-miR-29c-3p | 16 |
| hsa-miR-3065-3p | 16 |
| hsa-miR-3973 | 16 |
| hsa-miR-4642 | 16 |
| hsa-miR-3182 | 16 |
| hsa-miR-4646-3p | 16 |
| hsa-miR-16-5p | 16 |
| hsa-miR-548h-5p | 16 |
| hsa-miR-409-3p | 16 |
| hsa-miR-548i | 16 |
| hsa-miR-548ay-5p | 16 |
| hsa-miR-411-5p | 16 |
| hsa-miR-107 | 16 |
| hsa-miR-153-3p | 16 |
| hsa-miR-493-5p | 16 |
| hsa-miR-15a-5p | 16 |
| hsa-miR-4524a-5p | 16 |
| hsa-miR-195-5p | 16 |
| hsa-miR-299-5p | 16 |
| hsa-miR-7157-5p | 16 |
| hsa-miR-548p | 16 |
| hsa-miR-548y | 16 |
| hsa-miR-4711-3p | 16 |
| hsa-miR-497-5p | 16 |
| hsa-miR-6838-5p | 16 |
| hsa-miR-548ap-5p | 16 |
| hsa-miR-4524b-5p | 16 |
| hsa-miR-29a-3p | 16 |
| hsa-miR-3680-3p | 16 |
| hsa-miR-3912-5p | 16 |
| hsa-miR-548aq-5p | 16 |
| hsa-miR-449b-3p | 16 |
| hsa-miR-548ae-5p | 16 |
| hsa-miR-4288 | 16 |
| hsa-miR-545-3p | 16 |
| hsa-miR-603 | 16 |
| hsa-miR-548b-5p | 16 |
| hsa-miR-4495 | 16 |
| hsa-miR-548ab | 16 |
| hsa-miR-548am-5p | 16 |
| hsa-miR-4310 | 16 |
| hsa-miR-548o-5p | 16 |
| hsa-miR-5682 | 16 |
| hsa-miR-664b-3p | 16 |
| hsa-miR-6124 | 16 |
| hsa-miR-494-5p | 16 |
| hsa-miR-6835-3p | 16 |
| hsa-miR-548ak | 16 |
| hsa-miR-3120-3p | 16 |
| hsa-miR-548c-5p | 16 |
| hsa-miR-222-5p | 16 |
| hsa-miR-585-5p | 16 |
| hsa-miR-548j-5p | 16 |
| hsa-miR-1292-5p | 16 |
| hsa-miR-579-3p | 16 |
| hsa-miR-936 | 16 |
| hsa-miR-29b-3p | 16 |
| hsa-miR-424-5p | 16 |
| hsa-miR-548ar-5p | 16 |
| hsa-miR-548ad-5p | 16 |
| hsa-miR-548bb-5p | 16 |
| hsa-miR-548as-5p | 16 |
| hsa-miR-323b-5p | 16 |
| hsa-miR-548au-5p | 16 |
| hsa-miR-103a-3p | 16 |
| hsa-miR-1279 | 16 |
| hsa-miR-548a-5p | 16 |
| hsa-miR-548d-5p | 16 |
| hsa-miR-584-3p | 15 |
| hsa-miR-148a-3p | 15 |
| hsa-miR-197-3p | 15 |
| hsa-miR-7978 | 15 |
| hsa-miR-548n | 15 |
| hsa-miR-4501 | 15 |
| hsa-miR-6830-3p | 15 |
| hsa-miR-539-3p | 15 |
| hsa-miR-8054 | 15 |
| hsa-miR-4778-5p | 15 |
| hsa-miR-6875-3p | 15 |
| hsa-miR-628-3p | 15 |
| hsa-miR-374a-5p | 15 |
| hsa-miR-6509-5p | 15 |
| hsa-miR-548x-5p | 15 |
| hsa-miR-802 | 15 |
| hsa-miR-4422 | 15 |
| hsa-miR-632 | 15 |
| hsa-miR-302c-3p | 15 |
| hsa-miR-194-5p | 15 |
| hsa-miR-141-3p | 15 |
| hsa-miR-7-1-3p | 15 |
| hsa-miR-125b-2-3p | 15 |
| hsa-miR-6800-5p | 15 |
| hsa-miR-605-5p | 15 |
| hsa-miR-325 | 15 |
| hsa-miR-518a-5p | 15 |
| hsa-miR-548av-5p | 15 |
| hsa-miR-548f-5p | 15 |
| hsa-miR-298 | 15 |
| hsa-miR-633 | 15 |
| hsa-miR-647 | 15 |
| hsa-miR-548m | 15 |
| hsa-miR-548j-3p | 15 |
| hsa-miR-527 | 15 |
| hsa-miR-152-3p | 15 |
| hsa-miR-302d-3p | 15 |
| hsa-miR-4753-3p | 15 |
| hsa-miR-548aq-3p | 15 |
| hsa-miR-6754-3p | 15 |
| hsa-miR-5691 | 15 |
| hsa-miR-4724-3p | 15 |
| hsa-miR-8075 | 15 |
| hsa-miR-412-3p | 15 |
| hsa-miR-5590-3p | 15 |
| hsa-miR-4490 | 15 |
| hsa-miR-142-5p | 15 |
| hsa-miR-346 | 15 |
| hsa-miR-302e | 15 |
| hsa-miR-302a-3p | 15 |
| hsa-miR-548ah-3p | 15 |
| hsa-miR-103b | 15 |
| hsa-miR-548e-5p | 15 |
| hsa-miR-624-5p | 15 |
| hsa-miR-548am-3p | 15 |
| hsa-miR-374a-3p | 15 |
| hsa-miR-548l | 15 |
| hsa-miR-4760-3p | 15 |
| hsa-miR-548aw | 15 |
| hsa-miR-5696 | 15 |
| hsa-miR-335-3p | 15 |
| hsa-miR-6866-5p | 15 |
| hsa-miR-6515-3p | 15 |
| hsa-miR-5195-3p | 15 |
| hsa-miR-139-5p | 15 |
| hsa-miR-548ae-3p | 15 |
| hsa-miR-634 | 15 |
| hsa-miR-145-5p | 15 |
| hsa-miR-302b-3p | 15 |
| hsa-miR-7-2-3p | 15 |
| hsa-miR-4262 | 15 |
| hsa-miR-3671 | 15 |
| hsa-miR-548g-5p | 15 |
| hsa-miR-374b-5p | 15 |
| hsa-miR-548aj-5p | 15 |
| hsa-miR-675-3p | 15 |
| hsa-miR-6728-3p | 15 |
| hsa-miR-2052 | 15 |
| hsa-miR-4766-3p | 15 |
| hsa-miR-6805-3p | 15 |
| hsa-miR-6813-3p | 15 |
| hsa-miR-1256 | 15 |
| hsa-miR-200a-3p | 15 |
| hsa-miR-148b-3p | 15 |
| hsa-miR-548k | 15 |
| hsa-miR-4742-3p | 15 |
| hsa-miR-485-3p | 15 |
| hsa-miR-4684-3p | 15 |
| hsa-miR-4735-5p | 15 |
| hsa-miR-577 | 15 |
| hsa-miR-548aj-3p | 15 |
| hsa-miR-548x-3p | 15 |
| hsa-miR-758-5p | 15 |
| hsa-miR-582-3p | 15 |
| hsa-miR-6869-5p | 15 |
| hsa-miR-499a-3p | 14 |
| hsa-miR-451b | 14 |
| hsa-miR-371b-5p | 14 |
| hsa-miR-548ag | 14 |
| hsa-miR-548ba | 14 |
| hsa-miR-5002-5p | 14 |
| hsa-miR-6507-3p | 14 |
| hsa-miR-499b-3p | 14 |
| hsa-miR-570-5p | 14 |
| hsa-miR-550a-3p | 14 |
| hsa-miR-641 | 14 |
| hsa-miR-4652-3p | 14 |
| hsa-miR-548az-5p | 14 |
| hsa-miR-133b | 14 |
| hsa-miR-183-3p | 14 |
| hsa-miR-548t-5p | 14 |
| hsa-miR-5684 | 14 |
| hsa-miR-4677-3p | 14 |
| hsa-miR-616-5p | 14 |
| hsa-miR-3617-5p | 14 |
| hsa-miR-133a-3p | 14 |
| hsa-miR-548ai | 14 |
| hsa-miR-4527 | 14 |
| hsa-miR-373-5p | 14 |
| hsa-miR-3159 | 14 |
| hsa-miR-20a-3p | 14 |
| hsa-miR-200c-5p | 14 |
| hsa-miR-6503-5p | 14 |
| hsa-miR-595 | 14 |
| hsa-miR-4744 | 13 |
| hsa-miR-7108-5p | 13 |
| hsa-miR-885-5p | 13 |
| hsa-miR-3941 | 13 |
| hsa-miR-4682 | 13 |
| hsa-miR-4528 | 13 |
| hsa-miR-330-3p | 13 |
| hsa-miR-519b-3p | 12 |
| hsa-miR-519a-3p | 12 |
| hsa-miR-519c-3p | 12 |
| hsa-miR-4483 | 12 |
| hsa-miR-4531 | 12 |
| hsa-miR-3123 | 11 |
| hsa-miR-4305 | 11 |
| hsa-miR-624-3p | 11 |
| hsa-miR-627-5p | 11 |
| hsa-miR-3925-5p | 11 |
| hsa-miR-3911 | 10 |
| hsa-miR-4717-3p | 6 |
| hsa-miR-1303 | 5 |
| hsa-miR-100-3p | 5 |
| hsa-miR-12136 | 4 |
| hsa-miR-10522-5p | 4 |
| hsa-miR-1468-3p | 4 |
| hsa-miR-545-5p | 3 |
| hsa-miR-7851-3p | 3 |
| hsa-miR-12131 | 3 |
| hsa-miR-580-3p | 3 |
| hsa-miR-494-3p | 2 |
| hsa-miR-8066 | 2 |
| hsa-miR-4694-5p | 2 |
| hsa-miR-4738-3p | 2 |
| hsa-miR-4694-3p | 2 |
| hsa-miR-1267 | 2 |
| hsa-miR-5571-5p | 2 |
| hsa-miR-548b-3p | 2 |
| hsa-miR-219a-2-3p | 2 |
| hsa-miR-8485 | 2 |
| hsa-miR-3164 | 2 |
| hsa-let-7b-3p | 2 |
| hsa-miR-4704-3p | 2 |
| hsa-miR-6740-3p | 2 |
| hsa-let-7a-3p | 2 |
| hsa-miR-98-3p | 2 |
| hsa-miR-7154-5p | 2 |
| hsa-miR-380-3p | 2 |
| hsa-miR-4276 | 2 |
| hsa-miR-6820-3p | 2 |
| hsa-miR-644a | 2 |
| hsa-let-7f-1-3p | 2 |
| hsa-miR-4716-5p | 1 |
| hsa-miR-4267 | 1 |
| hsa-miR-7112-3p | 1 |
| hsa-miR-4516 | 1 |
| hsa-miR-493-3p | 1 |
| hsa-miR-6782-3p | 1 |
| hsa-miR-625-3p | 1 |
| hsa-miR-4311 | 1 |
| hsa-miR-507 | 1 |
| hsa-miR-4715-5p | 1 |
| hsa-miR-498-5p | 1 |
| hsa-miR-4295 | 1 |
| hsa-miR-597-3p | 1 |
| hsa-miR-515-5p | 1 |
| hsa-miR-150-5p | 1 |
| hsa-miR-4659b-5p | 1 |
| hsa-miR-324-3p | 1 |
| hsa-miR-5087 | 1 |
| hsa-miR-1297 | 1 |
| hsa-miR-649 | 1 |
| hsa-miR-1231 | 1 |
| hsa-miR-3619-5p | 1 |
| hsa-miR-3920 | 1 |
| hsa-miR-148a-5p | 1 |
| hsa-miR-301a-3p | 1 |
| hsa-miR-7852-3p | 1 |
| hsa-miR-6895-3p | 1 |
| hsa-miR-3137 | 1 |
| hsa-miR-6818-5p | 1 |
| hsa-miR-4659a-5p | 1 |
| hsa-miR-20b-3p | 1 |
| hsa-miR-520f-5p | 1 |
| hsa-miR-4540 | 1 |
| hsa-miR-6811-3p | 1 |
| hsa-miR-6753-3p | 1 |
| hsa-miR-1206 | 1 |
| hsa-miR-597-5p | 1 |
| hsa-miR-4758-5p | 1 |
| hsa-miR-5702 | 1 |
| hsa-miR-891a-3p | 1 |
| hsa-miR-4803 | 1 |
| hsa-miR-1257 | 1 |
| hsa-miR-199a-3p | 1 |
| hsa-miR-199b-3p | 1 |
| hsa-miR-301b-3p | 1 |
| hsa-miR-130a-3p | 1 |
| hsa-miR-4312 | 1 |
| hsa-miR-3163 | 1 |
| hsa-miR-490-3p | 1 |
| hsa-miR-520h | 1 |
| hsa-miR-3685 | 1 |
| hsa-miR-4293 | 1 |
| hsa-miR-214-3p | 1 |
| hsa-miR-5585-5p | 1 |
| hsa-miR-922 | 1 |
| hsa-miR-5003-3p | 1 |
| hsa-miR-1323 | 1 |
| hsa-miR-5579-3p | 1 |
| hsa-miR-646 | 1 |
| hsa-miR-302f | 1 |
| hsa-miR-581 | 1 |
| hsa-miR-7153-3p | 1 |
| hsa-miR-432-3p | 1 |
| hsa-miR-147a | 1 |
| hsa-miR-1236-3p | 1 |
| hsa-miR-8070 | 1 |
| hsa-miR-141-5p | 1 |
| hsa-miR-1322 | 1 |
| hsa-miR-4506 | 1 |
| hsa-miR-3688-3p | 1 |
| hsa-miR-891a-5p | 1 |
| hsa-miR-5582-3p | 1 |
| hsa-miR-5004-3p | 1 |
| hsa-miR-583 | 1 |
| hsa-miR-4724-5p | 1 |
| hsa-miR-1248 | 1 |
| hsa-miR-6790-5p | 1 |
| hsa-miR-1238-5p | 1 |
| hsa-miR-7107-3p | 1 |
| hsa-miR-3129-5p | 1 |
| hsa-miR-4291 | 1 |
| hsa-miR-892a | 1 |
| hsa-miR-567 | 1 |
| hsa-miR-10527-5p | 1 |
| hsa-miR-8081 | 1 |
| hsa-miR-6833-3p | 1 |
| hsa-miR-4670-3p | 1 |
| hsa-miR-4658 | 1 |
| hsa-miR-3617-3p | 1 |
| hsa-miR-5009-3p | 1 |
| hsa-miR-4777-3p | 1 |
| hsa-miR-130b-3p | 1 |
| hsa-miR-4711-5p | 1 |
| hsa-miR-506-3p | 1 |
| hsa-miR-557 | 1 |
| hsa-miR-34b-3p | 1 |
| hsa-miR-519e-5p | 1 |
| hsa-miR-5197-3p | 1 |
| hsa-miR-4788 | 1 |
| hsa-miR-5705 | 1 |
| hsa-miR-7152-5p | 1 |
| hsa-miR-548o-3p | 1 |
| hsa-miR-569 | 1 |
| hsa-miR-1179 | 1 |
| hsa-miR-3666 | 1 |
| hsa-miR-182-3p | 1 |
| hsa-miR-124-3p | 1 |
| hsa-miR-4733-3p | 1 |
| hsa-miR-520g-3p | 1 |
| hsa-miR-589-5p | 1 |
| hsa-miR-5701 | 1 |
| hsa-miR-454-3p | 1 |
| hsa-miR-7109-3p | 1 |
| hsa-miR-516a-5p | 1 |
| hsa-miR-367-5p | 1 |
| hsa-miR-4795-3p | 1 |
| hsa-miR-205-3p | 1 |
| hsa-miR-6513-3p | 1 |
| hsa-miR-29b-1-5p | 1 |
| hsa-miR-5692a | 1 |
| hsa-miR-8055 | 1 |
| hsa-miR-4279 | 1 |
| hsa-miR-4643 | 1 |
| hsa-miR-514a-5p | 1 |
| hsa-miR-4502 | 1 |
| hsa-miR-155-3p | 1 |
| hsa-miR-4263 | 1 |

**Supplementary Table 3: Virus encoded miRNAs present in genomes with their number of occurrences in each genome.**

| Virus encoded miRNAs present in most of the genomes | Number |
| --- | --- |
| hsa-miR-4471 | 33 |
| hsa-miR-3529-3p | 21 |
| hsa-miR-7-5p | 20 |
| hsa-miR-514a-3p | 20 |
| hsa-miR-4659a-5p | 18 |
| hsa-miR-6822-3p | 17 |
| hsa-miR-145-3p | 17 |
| hsa-miR-11400 | 16 |
| hsa-miR-548ao-3p | 16 |
| hsa-miR-582-5p | 16 |
| hsa-miR-6862-5p | 16 |
| hsa-miR-450a-2-3p | 16 |
| hsa-miR-122b-3p | 16 |
| hsa-miR-4502 | 16 |
| hsa-miR-1324 | 16 |
| hsa-miR-1248 | 16 |
| hsa-miR-3681-5p | 16 |
| hsa-miR-4764-5p | 16 |
| hsa-miR-506-3p | 16 |
| hsa-miR-877-3p | 16 |
| hsa-miR-548n | 16 |
| hsa-miR-584-5p | 16 |
| hsa-miR-6874-5p | 16 |
| hsa-miR-4761-3p | 16 |
| hsa-miR-676-3p | 16 |
| hsa-miR-1255b-5p | 16 |
| hsa-miR-3672 | 16 |
| hsa-miR-3682-5p | 16 |
| hsa-miR-609 | 16 |
| hsa-miR-4802-3p | 16 |
| hsa-miR-7161-3p | 16 |
| hsa-miR-4659b-3p | 16 |
| hsa-miR-4778-3p | 16 |
| hsa-miR-30b-5p | 16 |
| hsa-miR-545-3p | 16 |
| hsa-miR-4680-5p | 16 |
| hsa-miR-203a-5p | 16 |
| hsa-miR-6718-5p | 15 |
| hsa-miR-3675-3p | 15 |
| hsa-miR-140-5p | 15 |
| hsa-miR-627-3p | 15 |
| hsa-miR-196a-1-3p | 15 |
| hsa-miR-7705 | 15 |
| hsa-miR-1255a | 15 |
| hsa-miR-1273c | 15 |
| hsa-miR-515-5p | 15 |
| hsa-miR-892a | 15 |
| hsa-miR-133a-5p | 15 |
| hsa-miR-8062 | 15 |
| hsa-miR-125a-5p | 15 |
| hsa-miR-4521 | 15 |
| hsa-miR-892b | 15 |
| hsa-miR-122-5p | 15 |
| hsa-miR-1252-3p | 15 |
| hsa-miR-4801 | 15 |
| hsa-miR-3167 | 15 |
| hsa-miR-584-3p | 15 |
| hsa-miR-101-2-5p | 15 |
| hsa-miR-6507-3p | 15 |
| hsa-let-7i-5p | 15 |
| hsa-miR-186-5p | 15 |
| hsa-miR-186-3p | 15 |
| hsa-miR-4724-5p | 15 |
| hsa-miR-8088 | 15 |
| hsa-miR-6873-3p | 15 |
| hsa-miR-5197-3p | 15 |
| hsa-miR-194-5p | 14 |
| hsa-miR-3657 | 14 |
| hsa-miR-412-5p | 14 |
| hsa-miR-217-5p | 14 |
| hsa-miR-133a-3p | 14 |
| hsa-miR-4686 | 14 |
| hsa-miR-1539 | 14 |
| hsa-miR-1206 | 14 |
| hsa-miR-4804-3p | 14 |
| hsa-miR-936 | 14 |
| hsa-miR-133b | 14 |
| hsa-miR-4637 | 14 |
| hsa-miR-3609 | 14 |
| hsa-miR-4495 | 14 |
| hsa-miR-3975 | 14 |
| hsa-miR-8066 | 14 |
| hsa-miR-2115-5p | 13 |
| hsa-miR-3934-3p | 13 |
| hsa-miR-3908 | 13 |
| hsa-miR-506-5p | 13 |
| hsa-miR-20b-5p | 13 |
| hsa-miR-1255b-2-3p | 13 |
| hsa-miR-516b-5p | 13 |
| hsa-miR-1265 | 13 |
| hsa-miR-4308 | 13 |
| hsa-miR-4758-3p | 13 |
| hsa-miR-653-5p | 13 |
| hsa-miR-147b-5p | 12 |
| hsa-miR-3973 | 12 |
| hsa-miR-4640-5p | 12 |
| hsa-miR-624-5p | 12 |
| hsa-miR-6739-3p | 12 |
| hsa-miR-6781-3p | 12 |
| hsa-miR-548ba | 12 |
| hsa-miR-597-3p | 12 |
| hsa-miR-598-5p | 12 |
| hsa-miR-6506-3p | 12 |
| hsa-miR-3916 | 12 |
| hsa-miR-1911-5p | 12 |
| hsa-miR-221-5p | 12 |
| hsa-miR-4766-3p | 12 |
| hsa-miR-323a-5p | 11 |
| hsa-miR-12130 | 11 |
| hsa-miR-3617-5p | 11 |
| hsa-miR-1307-3p | 11 |
| hsa-miR-3116 | 11 |
| hsa-miR-4693-3p | 11 |
| hsa-miR-548b-5p | 11 |
| hsa-miR-3152-3p | 10 |
| hsa-miR-664a-3p | 10 |
| hsa-miR-15b-5p | 10 |
| hsa-miR-3934-5p | 10 |
| hsa-miR-5590-5p | 9 |
| hsa-miR-8082 | 9 |
| hsa-miR-3917 | 9 |
| hsa-miR-504-5p | 9 |
| hsa-miR-190b-5p | 9 |
| hsa-miR-551a | 9 |
| hsa-miR-513c-5p | 8 |
| hsa-miR-6768-3p | 8 |
| hsa-miR-6745 | 8 |
| hsa-miR-9851-5p | 8 |
| hsa-miR-4668-3p | 7 |
| hsa-miR-4307 | 7 |
| hsa-miR-8067 | 7 |
| hsa-miR-18a-5p | 7 |
| hsa-miR-548s | 7 |
| hsa-miR-136-5p | 7 |
| hsa-miR-1244 | 7 |
| hsa-miR-451b | 7 |
| hsa-miR-548a-3p | 7 |
| hsa-miR-483-3p | 6 |
| hsa-miR-561-5p | 6 |
| hsa-miR-4773 | 6 |
| hsa-miR-301b-3p | 6 |
| hsa-miR-561-3p | 6 |
| hsa-miR-544a | 6 |
| hsa-miR-17-3p | 6 |
| hsa-miR-5688 | 6 |
| hsa-miR-3128 | 6 |
| hsa-miR-3611 | 5 |
| hsa-miR-181d-5p | 5 |
| hsa-miR-744-3p | 5 |
| hsa-miR-7111-3p | 5 |
| hsa-miR-101-5p | 5 |
| hsa-miR-6857-3p | 5 |
| hsa-miR-3976 | 5 |
| hsa-miR-5009-3p | 5 |
| hsa-miR-6824-3p | 5 |
| hsa-miR-654-5p | 5 |
| hsa-miR-130a-3p | 5 |
| hsa-miR-3613-5p | 4 |
| hsa-miR-6514-5p | 4 |
| hsa-miR-548j-3p | 4 |
| hsa-miR-20a-3p | 4 |
| hsa-miR-3664-5p | 4 |
| hsa-miR-3664-3p | 4 |
| hsa-miR-4420 | 4 |
| hsa-miR-3155a | 4 |
| hsa-miR-448 | 4 |
| hsa-miR-5694 | 3 |
| hsa-miR-15a-5p | 3 |
| hsa-miR-4754 | 3 |
| hsa-miR-580-5p | 3 |
| hsa-miR-1193 | 3 |
| hsa-miR-145-5p | 3 |
| hsa-miR-137-3p | 3 |
| hsa-miR-3134 | 3 |
| hsa-miR-557 | 3 |
| hsa-miR-3159 | 3 |
| hsa-miR-380-5p | 3 |
| hsa-miR-7975 | 3 |
| hsa-miR-2682-5p | 2 |
| hsa-miR-8076 | 2 |
| hsa-miR-548q | 2 |
| hsa-miR-335-3p | 2 |
| hsa-miR-6838-3p | 2 |
| hsa-miR-3614-3p | 2 |
| hsa-miR-148b-3p | 2 |
| hsa-miR-513b-3p | 2 |
| hsa-miR-129-1-3p | 2 |
| hsa-miR-18b-5p | 2 |
| hsa-miR-497-5p | 2 |
| hsa-miR-6818-3p | 2 |
| hsa-miR-3059-3p | 2 |
| hsa-miR-15b-3p | 2 |
| hsa-miR-4703-5p | 2 |
| hsa-miR-33b-5p | 2 |
| hsa-miR-1304-3p | 2 |
| hsa-miR-4460 | 2 |
| hsa-miR-126-5p | 2 |
| hsa-miR-6715b-5p | 2 |
| hsa-miR-302d-5p | 2 |
| hsa-miR-3173-5p | 2 |
| hsa-miR-4753-3p | 2 |
| hsa-miR-6881-3p | 2 |
| hsa-miR-492 | 2 |
| hsa-miR-6772-5p | 2 |
| hsa-miR-4680-3p | 2 |
| hsa-miR-5088-5p | 2 |
| hsa-miR-6811-3p | 2 |
| hsa-miR-219a-1-3p | 2 |
| hsa-miR-548m | 2 |
| hsa-miR-548au-3p | 2 |
| hsa-miR-12127 | 2 |
| hsa-miR-767-5p | 2 |
| hsa-miR-19a-3p | 2 |
| hsa-miR-6770-5p | 2 |
| hsa-miR-655-3p | 1 |
| hsa-miR-6830-3p | 1 |
| hsa-miR-8059 | 1 |
| hsa-miR-4643 | 1 |
| hsa-miR-26b-3p | 1 |
| hsa-miR-3667-5p | 1 |
| hsa-miR-181a-2-3p | 1 |
| hsa-miR-1179 | 1 |
| hsa-miR-4712-5p | 1 |
| hsa-miR-6800-3p | 1 |
| hsa-miR-3064-5p | 1 |
| hsa-miR-2052 | 1 |
| hsa-miR-1261 | 1 |
| hsa-miR-4517 | 1 |
| hsa-miR-369-3p | 1 |
| hsa-miR-876-5p | 1 |
| hsa-miR-379-5p | 1 |
| hsa-miR-548ah-5p | 1 |
| hsa-miR-4483 | 1 |
| hsa-miR-3170 | 1 |
| hsa-miR-10397-5p | 1 |
| hsa-miR-3121-5p | 1 |
| hsa-miR-1305 | 1 |
| hsa-miR-2114-3p | 1 |
| hsa-miR-7152-5p | 1 |
| hsa-miR-3646 | 1 |
| hsa-miR-548ag | 1 |
| hsa-miR-489-3p | 1 |
| hsa-miR-3686 | 1 |
| hsa-miR-1288-5p | 1 |
| hsa-miR-7107-3p | 1 |
| hsa-miR-302a-3p | 1 |
| hsa-miR-617 | 1 |
| hsa-miR-548az-5p | 1 |
| hsa-miR-139-5p | 1 |
| hsa-miR-499b-3p | 1 |
| hsa-miR-3649 | 1 |
| hsa-miR-129-2-3p | 1 |
| hsa-miR-3122 | 1 |
| hsa-miR-550a-3p | 1 |
| hsa-miR-548ao-5p | 1 |
| hsa-miR-191-3p | 1 |
| hsa-miR-6769a-3p | 1 |
| hsa-miR-6772-3p | 1 |
| hsa-miR-548aq-5p | 1 |
| hsa-miR-6516-3p | 1 |
| hsa-miR-183-3p | 1 |
| hsa-miR-1270 | 1 |
| hsa-miR-216a-3p | 1 |
| hsa-miR-514a-5p | 1 |
| hsa-miR-223-5p | 1 |
| hsa-miR-5047 | 1 |
| hsa-miR-5707 | 1 |
| hsa-miR-10522-5p | 1 |
| hsa-miR-199a-5p | 1 |
| hsa-miR-153-3p | 1 |
| hsa-miR-8074 | 1 |
| hsa-miR-3158-3p | 1 |
| hsa-miR-4747-5p | 1 |
| hsa-miR-6854-3 | 1 |
| hsa-miR-132-5p | 1 |
| hsa-miR-548d-5p | 1 |
| hsa-miR-208b-3p | 1 |
| hsa-miR-4536-5p | 1 |
| hsa-miR-4781-3p | 1 |
| hsa-miR-520g-3p | 1 |
| hsa-miR-4789-3p | 1 |
| hsa-miR-4536-3p | 1 |
| hsa-miR-559 | 1 |
| hsa-miR-515-3p | 1 |
| hsa-miR-548u | 1 |
| hsa-miR-499a-5p | 1 |
| hsa-miR-548ae-5p | 1 |
| hsa-miR-6732-3p | 1 |
| hsa-miR-1468-5p | 1 |
| hsa-miR-6826-3p | 1 |
| hsa-miR-9500 | 1 |
| hsa-miR-5586-3p | 1 |
| hsa-miR-548ad-5p | 1 |
| hsa-miR-4445-3p | 1 |
| hsa-miR-603 | 1 |
| hsa-miR-548bb-3p | 1 |
| hsa-miR-6874-3p | 1 |
| hsa-miR-5692c | 1 |
| hsa-miR-10393-5p | 1 |
| hsa-miR-7161-5p | 1 |
| hsa-miR-154-5p | 1 |
| hsa-miR-548bb-5p | 1 |
| hsa-miR-7844-5p | 1 |
| hsa-miR-488-3p | 1 |
| hsa-miR-3911 | 1 |
| hsa-miR-132-3p | 1 |
| hsa-miR-3165 | 1 |
| hsa-miR-217-5p | 1 |
| hsa-miR-380-3p | 1 |
| hsa-miR-4301 | 1 |
| hsa-miR-371a-3p | 1 |
| hsa-miR-3129-3p | 1 |
| hsa-miR-4504 | 1 |
| hsa-miR-182-5p | 1 |
| hsa-miR-487b-5p | 1 |
| hsa-miR-1267 | 1 |
| hsa-miR-5701 | 1 |
| hsa-miR-579-3p | 1 |
| hsa-miR-556-5p | 1 |
| hsa-miR-6738-3p | 1 |
| hsa-miR-3156-5p | 1 |
| hsa-miR-3913-5p | 1 |
| hsa-miR-4676-3p | 1 |
| hsa-miR-7974 | 1 |
| hsa-miR-548aw | 1 |
| hsa-miR-12132 | 1 |
| hsa-miR-7153-5p | 1 |
| hsa-miR-802 | 1 |
| hsa-miR-181c-5p | 1 |
| hsa-miR-3154 | 1 |
| hsa-miR-3145-5p | 1 |
| hsa-miR-641 | 1 |
| hsa-miR-208a-3p | 1 |
| hsa-miR-6513-3p | 1 |
| hsa-miR-4671-5p | 1 |
| hsa-miR-5589-3p | 1 |
| hsa-miR-6792-5p | 1 |
| hsa-miR-624-3p | 1 |
| hsa-miR-335-5p | 1 |
| hsa-miR-199b-5p | 1 |
| hsa-miR-6506-5p | 1 |
| hsa-miR-548j-5p | 1 |
| hsa-miR-5692b | 1 |
| hsa-miR-4663 | 1 |
| hsa-miR-1912-5p | 1 |
| hsa-miR-4782-5p | 1 |
| hsa-miR-5008-5p | 1 |
| hsa-miR-26a-1-3p | 1 |
| hsa-miR-4753-5p | 1 |
| hsa-miR-12133 | 1 |
| hsa-miR-6128 | 1 |
| hsa-miR-511-5p | 1 |
| hsa-miR-337-3p | 1 |
| hsa-miR-12123 | 1 |
| hsa-miR-130b-5p | 1 |
| hsa-miR-548at-3p | 1 |
| hsa-miR-4679 | 1 |
| hsa-miR-4445-5p | 1 |
| hsa-miR-550b-2-5p | 1 |
| hsa-miR-125b-1-3p | 1 |
| hsa-miR-4662a-5p | 1 |
| hsa-miR-1197 | 1 |
| hsa-miR-2114-5p | 1 |
| hsa-miR-3158-5p | 1 |
| hsa-miR-6755-3p | 1 |
| hsa-miR-548ay-5p | 1 |
| hsa-miR-214-3p | 1 |
| hsa-miR-3913-3p | 1 |
| hsa-miR-384 | 1 |
| hsa-miR-6832-5p | 1 |
| hsa-miR-12128 | 1 |
| hsa-miR-3942-3p | 1 |
| hsa-miR-511-3p | 1 |
| hsa-miR-5579-3p | 1 |
| hsa-miR-6885-5p | 1 |
| hsa-miR-454-5p | 1 |
| hsa-miR-942-5p | 1 |
| hsa-miR-548y | 1 |
